# Plasma metals as potential biomarkers in dementia: a case–control study in patients with sporadic Alzheimer’s disease

**DOI:** 10.1007/s10534-018-0089-3

**Published:** 2018-03-07

**Authors:** Jingshu Xu, Stephanie J. Church, Stefano Patassini, Paul Begley, Katherine A. B. Kellett, Emma R. L. C. Vardy, Richard D. Unwin, Nigel M. Hooper, Garth J. S. Cooper

**Affiliations:** 1 Centre for Advanced Discovery and Experimental Therapeutics (CADET), Division of Cardiovascular Sciences, School of Medical Sciences, Faculty of Biology, Medicine and Health, The University of Manchester, Manchester, UK; 2 Central Manchester University Hospitals NHS Foundation Trust, Manchester Academic Health Science Centre, Manchester, UK; 3 Division of Neuroscience and Experimental Psychology, School of Biological Sciences, Faculty of Biology, Medicine and Health, The University of Manchester, Manchester Academic Health Science Centre, Manchester, UK; 4 Salford Royal NHS Foundation Trust, Manchester Academic Health Science Centre, Salford, UK; 5 School of Biological Sciences and Maurice Wilkins Centre for Molecular Biodiscovery, Faculty of Science, University of Auckland, Auckland, New Zealand; 6 Rm 3.08, Core Technology Facility, Division of Cardiovascular Sciences, Faculty of Biology, Medicine and Health, The University of Manchester, Grafton Street, Manchester, M13 9NT UK

**Keywords:** Alzheimer’s disease, Dementia, Neurodegeneration, Human plasma, Metal biomarker, Plasma-zinc levels

## Abstract

**Abstract:**

Sporadic Alzheimer’s disease (AD) is a neurodegenerative disorder that causes the most prevalent form of age-related dementia but its pathogenesis remains obscure. Altered regulation of metals, particularly pan-cerebral copper deficiency, and more regionally-localized perturbation of other metals, are prominent in AD brain although data on how these CNS perturbations are reflected in the peripheral bloodstream are inconsistent to date. To assess the potential use of metal dysregulation to generate biomarkers in AD, we performed a case–control study of seven essential metals and selenium, measured by inductively coupled plasma mass-spectrometry, in samples from AD and matched control cases. Metals were sodium, potassium, calcium, magnesium, iron, zinc, and copper. In the whole study-group and in female participants, plasma metal levels did not differ between cases and controls. In males by contrast, there was moderate evidence that zinc levels trended towards increase in AD [10.8 (10.2–11.5)] µmol/L, mean (± 95% CI; *P *= 0.021) compared with controls [10.2 (9.6–10.4)]. Thus alterations in plasma zinc levels differed between genders in AD. In correlational analysis, there was evidence for an increased number of ‘strong’ metal co-regulations in AD cases and differential co-modulations of metal pairs: copper-sodium (*R*_control_ = − 0.03, *R*_AD_ = 0.65; *P *= 0.009), and copper-calcium (*R*_control_ = − 0.01, *R*_AD_ = 0.65; *P *= 0.01) were significant in AD males, potentially consistent with reported evidence for dysregulation of copper in severely damaged brain regions in AD. In conclusion, our data suggest that the measurement of metals co-regulation in plasma may provide a useful representation of those metal perturbations taking place in the AD brain and therefore might be useful as plasma-based biomarkers.

**Graphical Abstract:**

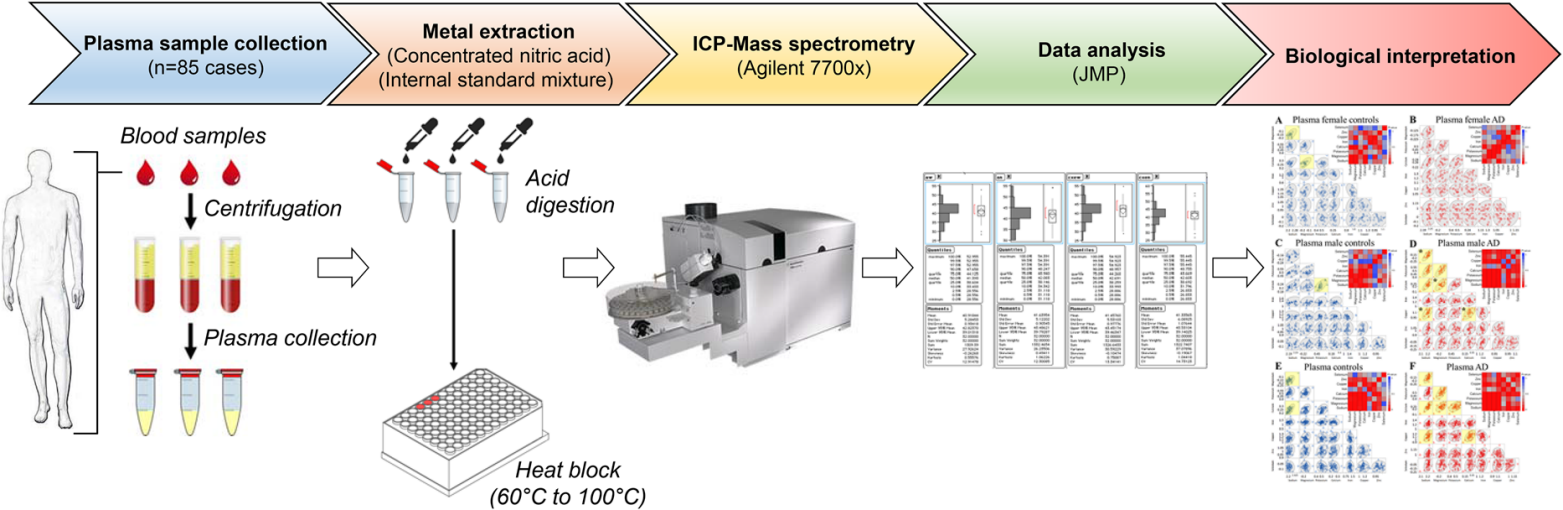

## Introduction

Sporadic Alzheimer’s disease (AD), which has no known genetic basis, is the predominant cause of age-related dementia globally (Ferri et al. 2005). Dementia has been predicted to affect around 136 million of the world-wide population by 2050 (Prince et al. 2013) but at present there are no treatments with proven disease-modifying efficacy (Citron 2010). Therefore, AD is the largest unmet medical need in neurology and the associated socio-economic burden on our healthcare system is already huge and expected to grow dramatically in the future.

Major neuropathological hallmarks of AD include the presence of amyloid-β (Aβ) plaques and neurofibrillary tangles (NFTs) formed from tau protein, and synaptic loss. Proposed underlying mechanisms for AD pathogenesis include changes in the molecular regulation of amyloid precursor protein (APP) metabolism, oxidative stress, impaired energetics, mitochondrial dysfunction, inflammation, membrane lipid dysregulation and neurotransmitter pathway disruption (Kaddurah-Daouk et al. 2013). Defective regulation of metal homeostasis is also believed to play a significant role in the pathogenesis of AD, however, there is no therapy targeting metal homeostasis registered for the treatment of AD at present (Ayton et al. 2013; Bush 2013; Sastre et al. 2015; Squitti 2012).

The human brain is a highly oxidative organ that contains abundant antioxidants, which can counteract the detrimental actions of reactive oxygen species (ROS) produced during the reaction of redox-active metals and molecular oxygen (Smith et al. 2007). Aβ peptide can reportedly generate ROS in the presence of certain transition metals (Huang et al. 1999, 2004; Smith et al. 2007), which not only affect the synthesis, degradation and clearance of Aβ but also participate in the aggregation of both Aβ and NFT in the brain (Adlard and Bush 2006; Sastre et al. 2015). Acting in reverse, there is some evidence that both APP and Aβ might modulate brain-metal homoeostasis. Furthermore, as an integral component of many metalloenzymes, metals modulate the normal function of numerous enzymes, processes and pathways via catalysis (Roberts et al. 2012).

In the AD research community, there is common agreement that advancements towards an effective therapy are hampered by the absence of robust and sensitive biomarkers that are needed as diagnostic, prognostic, predictive and pharmacodynamic-pharmacokinetic measurements to rapidly take decisions in the clinic and to quickly monitor the efficacy of disease-modifying therapies in clinical trials (Fiandaca et al. 2014).

Recently, systematic screening of plasma metal levels by inductively-coupled-plasma mass-spectrometry (ICP-MS) revealed the presence of altered metal metabolism in medical conditions involving the CNS (Nahan et al. 2017). In addition, the authors suggested that plasma metals might represent a good biomarker to gather diagnostic information in CNS-associated disorders. Here, in order to establish useful and accessible biomarkers of AD status, we have chosen to monitor events that are likely linked to fundamental features of AD pathogenesis or mechanisms underlying neurodegeneration in AD. As defects in metal homeostasis have been widely established as a major feature of AD pathogenesis, we investigated whether the aberrant metal metabolism reported in AD brain is also reflected in an easily accessible source such as plasma (Akatsu et al. 2012; Ramos et al. 2014; Sastre et al. 2015; Xu et al. 2017). Whether it is possible to detect any dysregulation in metal levels in the plasma of AD cases, who undergo brain-metal perturbations (Xu et al. 2017), is critical for determining whether measurements incorporating metal levels might serve as diagnostic, prognostic, progression and pharmacodynamic/pharmacokinetic biomarkers of AD in the future.

Hence, the concentrations of elements, including the metals sodium, Na; magnesium, Mg; potassium, K; calcium, Ca; iron, Fe; copper, Cu; and zinc, Zn; and the metalloid selenium, Se were determined by ICP-MS in plasma from 42 patients with AD and in 43 controls with no evidence of impaired memory or clinical evidence for neurological disorder.

## Materials and methods

### Ethics

We performed this case–control study under a protocol approved by the Leeds Teaching Hospitals NHS Trust Research Ethics Committee. All procedures accorded with the ethics committee guidelines with informed consent from all participants or families of patients as appropriate.

### Patient selection

Patients and controls without cognitive impairment were respectively recruited (Vardy et al. 2009) through memory clinics in Leeds and Dewsbury (England) and the Leeds Family Health Services Authority day hospitals and elderly medicine outpatient clinics in the Leeds area. All were of European Caucasian background and gave written informed consent (consent from relatives of the AD-patients was provided, where appropriate). Diagnosis of probable AD was made in accordance with international diagnostic criteria (National Institute of Neurological and Communicative Disorders and Stroke–Alzheimer’s Disease and Related Disorders Association Work Group: NINCDS-ADRDA) (McKhann et al. 1984). All participants underwent a standardised clinical evaluation: medical history, fasting plasma glucose (FPG) and HbA1c (a measure of chronic glycaemia), and cognitive function assessment by Mini-Mental State Examination (MMSE). AD samples were selected by excluding patients with diagnosed T1D or T2D (including those taking insulin). Samples were then selected from the whole-study population for whom required measurements (FPG, HbA1c) were available (Vardy et al. 2007). The resulting 42 AD-patients were then age- and gender-matched to 43 controls (Table 1).

**Table 1:** Characteristics of participants in this study

Characteristic	AD	Control	*P* value
Number	42	43	–
Male (%)	52.4	53.5	NS
Age (years)	78.2 ± 1.3	78.1 ± 1.1	NS
MMSE score	21 (11–25)	29 (27–30)	< 0.001
ApoE4 allele-positive (%)	71.4	32.6	< 0.001
Fasting plasma glucose (mmol/l)	5.0 (3.8–10.6)	5.1 (4.2–8.9)	NS
Serum HbA1c^a^	5.6 (5.1–7.8)	5.7 (5.1–8.4)	NS

^**a**^Hemoglobin A1c

### Sample digestion

Plasma samples of 50 μL were digested in 2-mL microcentrifuge tubes (Eppendorf) as described below. Detailed method descriptions are available (Xu et al. 2017). Reactions were performed in concentrated nitric acid (A509 Trace Metal Grade; Fisher, Loughborough, UK) to which had been added 5% (v/v) Agilent Internal Standard mixture (5183-4681; Agilent Technologies, Cheadle, UK). This internally standardised acid was also used at appropriate dilutions to provide rinse and calibration solutions, at 2% (v/v) final nitric acid concentration.

Calibration solutions were produced by appropriate dilutions of Environmental Calibration Standard (Agilent 5183-4688). Acid digestion was carried out using an ‘open-vessel’ method. The tube lids were punctured to prevent pressure build up, and 0.2 ml standard-containing nitric acid added. Tubes were then inserted into a “Dri-block” heater, which was initially at room temperature. In parallel, 50-µl aliquots of NIST SRM 1950 plasma (Standard Reference Material; National Institute of Standards and Technology, Gaithersburg, MD), were processed in the same way. Tubes with standard-containing acid but no sample were also processed in each batch to provide “digestion” blanks. Temperature was then set to 60 °C and the block switched on. After 30 min, the set temperature was increased to 100 °C, and digestion continued for a further 210 min. After digestion, the tubes were allowed to cool overnight.

Aliquots of 100 μL were taken from each digestion solution and added to 15-mL Falcon tubes (525–0629, VWR, Lutterworth, UK) containing 5 mL LC–MS grade water, to produce solutions for analysis at a final nitric acid concentration of 2% (v/v).

### Metal measurements

Tissue metal concentrations were measured using an Agilent 7700× ICP-MS spectrometer equipped with a MicroMist nebulizer (Glass Expansion, Melbourne, Australia) and a Scott double-pass spray chamber. Sample introduction was performed using an Agilent Integrated autosampler (I-AS) with helium as the collision gas. A multi-element method including all elements present in the calibration solution was applied, as previously reported by our group (Church et al. 2015; Xu et al. 2017). Calibration solutions were produced by appropriate dilutions of Environmental Calibration Standard (Agilent 5183-4688). An intermediate concentration from this calibration series was used throughout as a periodic quality control sample. Instrument and digestion blanks were also interspersed throughout the experiment and the detection limit for each element determined by comparison of calibration samples and blanks. Scandium was used as the internal standard for all elements except Zn and Se, where germanium was used. Two collision-cell gas modes were applied: all elements were analysed in helium mode (5.0 ml/min helium), except for Se which was analysed in high-energy helium mode (10 ml/min helium). Germanium internal standard was analysed in both modes. Mode selection followed Agilent recommendations to minimise interference for measured elements by e.g. isobaric cluster ions. Integration times were: 0.1 s for Na, Mg, K and Ca; 0.3 s for Cu and Zn; 0.01 s for Fe; and 3 s for Se. NIST SRM plasma samples were also analysed within this experiment to check for precision of our measurements when compared to the certified values.

### Statistical methods

The analysis for case–control study group characteristics was performed using SPSS Statistics 22 (IBM). Distributions of variables were tested for skewness using the Kolmogorov–Smirnov Goodness-Of-Fit test, for 42 AD participants and 43 matched controls (Table 1). Parametric data are presented as mean ± standard error of the mean (SEM), and non-parametric data as median (range). Comparisons between AD and control groups were performed using an Independent-Samples *t* test for parametric, and the Mann–Whitney *U* test for non-parametric data. Pearson’s χ^2^ analysis was performed on categorical data.

ICP-MS data were exported to Microsoft Excel and log-transformed for statistical analysis. Means (± 95% CI) of the log-transformed data were calculated and the significance of between-group differences was examined by unpaired *t*-tests with Welch’s correction to allow for unequal variances and sample sizes. Means (± 95% CI) were back-transformed to reflect the actual concentrations of elements. Statistical calculations were performed using GraphPad v6.04 (Prism; La Jolla, CA). *P* values of < 0.05 have been considered significant, and those of 0.05 ≤ P < 0.10 have also been tabulated. For correlation analyses, serial pairwise correlations were generated between the reported metal abundances (log_10_ transformed) using the statistical software JMP^®^ (Version 12.0.0; SAS Institute, Cary, NC). The correlations generated were visually depicted using scatter plot matrices and those considered as “strong” (Pearson coefficient *R* > 0.5 (or < − 0.5) and *P* values < 0.001) were further investigated. The statistical significance of differences in correlation coefficients between strong correlations in controls and AD groups was determined by Fisher r-to-z transformation and resulting *P* values < 0.05 (two-tailed) were considered significant.

## Results and discussion

In this study, we measured concentrations of seven essential metals and the metalloid, Se, in plasma samples from 42 cases with AD and 43 matched control participants (Table 1). We matched participants in this case–control study for gender and age (Table 1). Consistent with expectation, measured cognitive function (MMSE scores) was significantly lower in the AD group than controls whereas the ApoE4 allele was more prevalent. Levels of FPG and serum HbA1c were equivalent between groups. There was no evidence for elevated rates of undiagnosed T2D or impaired glucose tolerance (IGT)/impaired fasting glucose (IFG) in this group of British patients with sporadic AD (Table 1). Therefore, the current study compares plasma metal levels in AD cases representative of sporadic AD with appropriately matched controls.

We rigorously controlled the application of the ICP-MS methodology and have a high degree of confidence in the robustness of our data. For example, we included NIST SRM plasma samples in our study as previously described (Xu et al. 2017) and verified that measurements were within the ± 5% difference range compared to the certified values, indicating the precision of the methodology applied in this study.

**Fig. 1:**
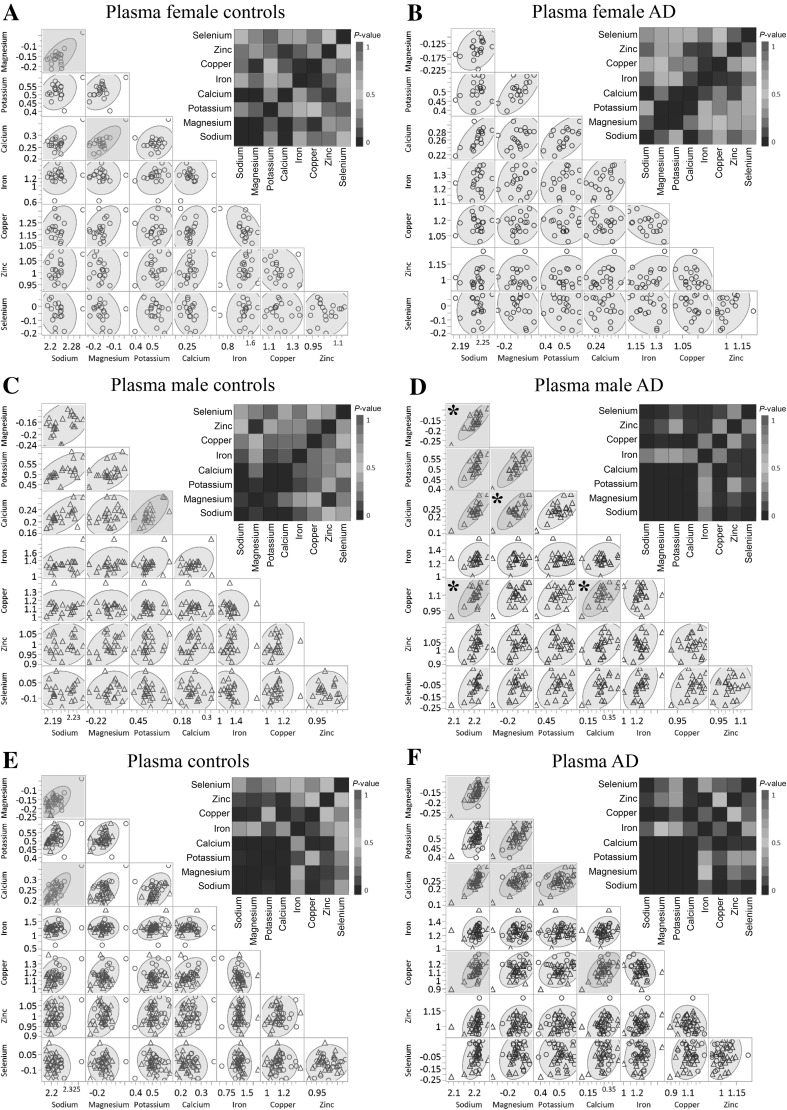
Correlations between metal levels in the plasma of AD cases and controls. Scatter plots of metal abundance correlations are shown for each metal measured in control (blue) and AD (red) plasma in females (**a**, **b**), male (**c**, **d**) and combined sexes (**e**, **f**). Shown are *P* values corresponding to each metal-pair, with correlations as a gradient colour in the heat map for each gender group. All the strong correlations (*R *> 0.5 (or < − 0.5) and *P* values of < 0.001) are highlighted in yellow and, for each correlation, grey ellipsoids represent the 95% confidence interval. Note that the metal co-regulations were only perturbed in the male AD cases whereas values were unaffected in females or combined groupings. *Significantly different correlations (Fisher r-to-z transformation *P* values < 0.05) between control and AD participants in the male group. Legend: control males (blue triangles); control females (blue circles); AD males (blue circles); AD females (red circles). (Color figure online)

We analysed the female and male datasets separately, and then combined them, in order to highlight possible effects of gender in control and AD participants (Table 2, Fig. 1). In the female patients, none of the eight measured elements differed in their plasma concentrations between the AD and control groups (*P* = 0.021; Table 2). However, in male patients, there was modest evidence that Zn trended towards increase in the AD group compared to the control group (Table 2). This might be consistent with previously-reported elevations in levels of Zn in AD brain (Schrag et al. 2011), and possibly also with enrichment of Zn in and around amyloid plaques (Lovell et al. 1998). However, reports concerning elevations of Zn in AD brain are not uniform. For example, in a recent metallomic study, brain-Zn levels in AD were moderately elevated in only three of seven regions studied: entorhinal cortex, middle temporal gyrus, and cerebellum (Xu et al. 2017). By contrast, they were similar to control values in other regions known to contain high levels of amyloid plaques in AD. Thus, the relationship between bulk regional Zn concentrations and Zn in amyloid plaques is not entirely clear. Since multi-regional studies of brain metal content are sparse, it is difficult to reach firm conclusions concerning the occurrence or significance of elevated brain-Zn levels in AD.

**Table 2:** Plasma metal concentrations in groups of females, males and combined sexes

Element (reference isotope)	Concentration unit	Groups	Control	AD	*P* value
Na (^23^Na)	mmol/L	F	170 (166–175)	167 (164–169)	NS
		M	160 (158–162)	162 (156–167)	NS
		F & M	165 (162–167)	164 (161–167)	NS
Mg (^24^Mg)	mmol/L	F	0.70 (0.67–0.73)	0.70 (0.69–0.74)	NS
		M	0.70 (0.66–0.71)	0.70 (0.68–0.73)	NS
		F & M	0.70 (0.67–0.71)	0.70 (0.69–0.73)	NS
K (^39^K)	mmol/L	F	3.3 (3.2–3.5)	3.2 (3.0–3.4)	NS
		M	3.3 (3.1–3.4)	3.3 (3.1–3.4)	NS
		F & M	3.3 (3.2–3.4)	3.3 (3.1–3.4)	NS
Ca (^44^Ca)	mmol/L	F	1.9 (1.8–1.9)	1.8 (1.8–1.9)	NS
		M	1.7 (1.6–1.7)	1.8 (1.7–1.8)	0.087
		F & M	1.8 (1.7–1.8)	1.8 (1.7–1.8)	NS
Fe (^56^Fe)	μmol/L	F	17.6 (14.5–21.3)	17.8 (16.3–19.5)	NS
		M	20.1 (16.9–24.1)	18.4 (16.7–20.4)	NS
		F & M	18.9 (16.6–21.5)	18.1 (17.0- 19.4)	NS
Cu (^63^Cu)	μmol/L	F	15.9 (14.6–17.3)	14.5 (13.3–15.8)	NS
		M	13.2 (12.1–14.3)	12.7 (11.7–13.9)	NS
		F & M	14.4 (13.5–15.3)	13.5 (12.7–14.4)	NS
Zn (^66^Zn)	μmol/L	F	10.2 (9.8–10.7)	10.5 (9.5–11.6)	NS
		M	10.0 (9.6–10.4)	10.8 (10.2–11.5)	0.021*
		F & M	10.1 (9.8–10.4)	10.7 (10.1–11.2)	0.075
Se (^78^Se)	μmol/L	F	0.90 (0.84–0.98)	1.00 (0.89–1.05)	NS
		M	0.90 (0.86–1.01)	0.90 (0.81–0.95)	NS
		F & M	0.90 (0.87–0.97)	0.90 (0.86–0.97)	NS

Data are means (± 95% CI); *P* values for significance of between-group differences were calculated by applying Welch’s modified *t* tests to metal measurements from plasma samples from female patients (F) [control (n = 20) and AD (n = 19)], male patients (M) [control (n = 23) and AD (n = 23)], and all patients combined (F & M) [control (n = 43) and AD (n = 42)]

*Significant (*P* values < 0.05)

In addition, the observation of elevated serum-Zn levels in males but not females with AD is an intriguing finding. Some workers have suggested that altered Zn homeostasis could play a significant role in AD pathogenesis, since it can clearly interact with APP and Aβ, and reportedly participates in APP processing, and aggregation and clearance of Aβ in the AD-brain. Data strongly support the enrichment of Zn in the core of Aβ plaques, a process that has been suggested to disrupt Zn homeostasis in brain regions important for memory generation and vulnerable to AD pathogenesis (Craddock et al. 2012; Miller et al. 2006). However, we note that interventions aimed at ameliorating alterations in Zn homeostasis have no accepted role in the management of AD at the time of writing.

In our combined analyses that included separate analysis of data from both genders, none of the measured metals showed statistically significant between-group concentration differences (Table 2). Therefore, our results suggest that alterations in plasma metal levels in association with AD may be inconsistent between genders. In line with our observations, a previous study concluded that metal levels are not consistently altered in plasma of AD participants, except for an elevated abundance of mercury (Gerhardsson et al. 2008). Other studies have reported decreased plasma levels of Mg, Cu, Zn, Fe and Se (Vural et al. 2010) and serum levels of Zn (Huang et al. 2013) in patients with AD compared with controls using different approaches, but the findings of our current study are not consistent with these prior reports, perhaps due to the minor differences of method employed. However, we would note that the evidence generated from our study is strongly supported by the methods employed and the use of the NIST SRM as a control to verify the efficacy of the digestion and metal recovery and measurements processes [for further data concerning the performance of our methods, see (Xu et al. 2017)].

We also performed an exploratory pairwise correlational analysis to ascertain how the measured metals might be differently co-regulated between controls and cases. Firstly, the number of strong correlations (*R* > 0.5 (or < − 0.5) and *P* values < 0.001) was determined for each group (Table 3, Fig. 1). We observed a higher number of strong correlations in the all-AD group (~ 18% increase; Table 3), a finding possibly consistent with a systemic alteration of metal co-regulation in these patients. Notably, when we divided the groups by gender, only the AD-male group showed an increase in strong correlations (~ 21.5% increase). In addition, in the male group there were significant changes between pairwise correlations in metal levels between the controls and cases. Significant differences included changes in the following metal–metal pairs: magnesium-sodium (*R*_control_ = 0.41, *R*_AD_ = 0.84; *P* = 0.02); calcium-magnesium (*R*_control_ = 0.28, *R*_AD_ = 0.73; *P* = 0.04); copper-sodium (*R*_control_ = − 0.03, *R*_AD_ = 0.65; *P *= 0.009); and copper-calcium (*R*_control_ = − 0.02, *R*_AD_ = 0.65; *P *= 0.01). These copper-associated correlations were the most significant differences observed in AD-male participants when compared to controls (Fig. 1a–f). Interestingly, in another study in patients with AD, substantively elevated sodium levels were present in all three severely damaged brain regions assessed: hippocampus, entorhinal cortex, and middle temporal gyrus. Copper was markedly decreased in these regions as well, but also in four other brain regions studied (Xu et al. 2017). Therefore, there is some evidence for a copper-sodium interaction in the brain of patients with AD, which may be related to the finding of a sodium-copper correlation in the plasma.

**Table 3:** Number of correlations for each group

Gender group^a^	Strong correlations controls	Strong correlations AD	Shared correlations controls/AD
Female (F)	2 (7.1%)	0 (0%)	0 (0%)
Male (M)	1 (3.6%)	7 (25.0%)	0 (0%)
Combined (F & M)	2 (7.1%)	7 (25.0%)	2 (7.1%)

^a^In each group, 28 correlations were tested for significance

T2D has strong epidemiological ties with AD, and T2D is known to cause significant perturbations in the systemic homeostasis of numerous essential metals (Cooper et al. 2005). It was therefore important to eliminate it from both groups in the current study, to exclude any possible confounding effect from participants with known T2D or those with undiagnosed disease. We did this by history, and screening all potential participants for T2D using FPG and HbA1c measurements. This is a precaution that should be undertaken in future studies of systemic metal regulation in AD, in order to avoid a possible confounding effect by untoward inclusion of patients with T2D, particularly given reports of increased prevalence of T2D in populations of patients with AD (Janson et al. 2004). In the current study therefore, alterations in metal homeostasis detected in participants with AD were not due to possible T2D.

While plasma levels of individual metals are of limited informational value here, how these metals are differentially co-regulated may represent a more useful measurement for further investigation as potential diagnostic biomarker of AD status. Likewise, other groups have indicated correlation coefficient matrix-assessed correlations as a suitable method to enable biomarker discovery in CNS-associated disorders (Nahan et al. 2017). Indeed, correlation analyses may assist in unmasking global dysregulation of systemic metal homeostasis in AD in a more comprehensive way as many of the pathways where metals play distinctive roles are mutually interconnected. As just one example, Cu plays a major role in the regulation of systemic uptake and distribution of Fe (Nittis and Gitlin 2002). Therefore, we suggest that larger scale studies are needed to verify the alteration of plasma metal levels in AD and we indicate the monitoring of co-regulated plasma metals as a relevant measurement to be tested as potential diagnostic biomarker in AD.

Finally, our results have significance for guiding future investigations on the utility of plasma metals as potential peripheral biomarkers to track rapidly progression or severity in subjects with AD, and possibly to predict disease onset before clinical and neurological symptoms are clearly manifested. In addition, plasma metals might be relevant as pharmacodynamic/pharmacokinetic biomarkers to facilitate clinical studies involving metal chelation treatment in AD.

## Abbreviations

Aβ: Amyloid-beta
AD: Alzheimer’s disease
APP: Amyloid precursor protein
BBB: Blood-brain barrier
CI: Confidence interval
FPG: Fasting plasma glucose
HbA1c: Hemoglobin A1c
ICP-MS: Inductively-coupled-plasma mass spectrometry
LC-MS: Liquid-chromatography mass-spectrometry
MMSE: Mini-mental state examination
NFT: Neurofibrillary tangle
NIST: National institute of standards and technology
ROS: Reactive oxygen species
SRM: Standard reference material
T2D: Type-2 diabetes
